# *N*-Cyclo­hexyl­benzamide

**DOI:** 10.1107/S1600536810022609

**Published:** 2010-06-18

**Authors:** Islam Ullah Khan, Rashid Javaid, Shahzad Sharif, Edward R. T. Tiekink

**Affiliations:** a Materials Chemistry Laboratory, Department of Chemistry, Government College, University, Lahore 54000, Pakistan; b Department of Chemistry, University of Malaya, 50603 Kuala Lumpur, Malaysia

## Abstract

The structure of the title compound, C_13_H_17_NO, features an *anti* disposition of the N—H and carbonyl groups. The amide group is twisted with respect to the benzene ring [N–C(=O)–C–C torsion angle = −30.8 (4)°]. In the crystal, *C*(4) chains propagating in [100] are formed by inter­molecular N–H⋯O hydrogen bonds. Weak C—H⋯π inter­actions link the chains into sheets.

## Related literature

For biological applications of benzamides, see: Clark *et al.* (1988 ▶); Leander *et al.* (1988 ▶); Diouf *et al.* (1997 ▶).
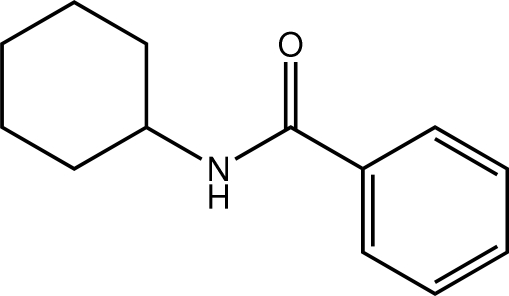


## Experimental

### 

#### Crystal data

C_13_H_17_NO
*M* *_r_* = 203.28
Monoclinic, 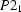

*a* = 5.2372 (3) Å
*b* = 6.5841 (4) Å
*c* = 16.6029 (12) Å
β = 91.176 (2)°
*V* = 572.38 (6) Å^3^
*Z* = 2
Mo *K*α radiation
μ = 0.07 mm^−1^
*T* = 293 K
0.28 × 0.17 × 0.12 mm

#### Data collection

Bruker APEXII CCD diffractometer
5479 measured reflections
1423 independent reflections
1105 reflections with *I* > 2σ(*I*)
*R*_int_ = 0.033

#### Refinement

*R*[*F* ^2^ > 2σ(*F* ^2^)] = 0.045
*wR*(*F* ^2^) = 0.163
*S* = 1.07
1423 reflections
140 parameters
1 restraint
H atoms treated by a mixture of independent and constrained refinement
Δρ_max_ = 0.22 e Å^−3^
Δρ_min_ = −0.21 e Å^−3^

### 

Data collection: *APEX2* (Bruker, 2007 ▶); cell refinement: *SAINT* (Bruker, 2007 ▶); data reduction: *SAINT*; program(s) used to solve structure: *SHELXS97* (Sheldrick, 2008 ▶); program(s) used to refine structure: *SHELXL97* (Sheldrick, 2008 ▶); molecular graphics: *ORTEP-3* (Farrugia, 1997 ▶) and *DIAMOND* (Brandenburg, 2006 ▶); software used to prepare material for publication: *publCIF* (Westrip, 2010 ▶).

## Supplementary Material

Crystal structure: contains datablocks global, I. DOI: 10.1107/S1600536810022609/hb5496sup1.cif

Structure factors: contains datablocks I. DOI: 10.1107/S1600536810022609/hb5496Isup2.hkl

Additional supplementary materials:  crystallographic information; 3D view; checkCIF report

## Figures and Tables

**Table 1:** Hydrogen-bond geometry (Å, °) *Cg*1 is the centroid of the C2–C7 ring.

*D*—H⋯*A*	*D*—H	H⋯*A*	*D*⋯*A*	*D*—H⋯*A*
N1—H1*n*⋯O1^i^	0.80 (3)	2.32 (3)	3.065 (3)	157 (3)
C13—H13a⋯*Cg*1^ii^	0.97	2.82	3.722 (4)	154
C5—H5⋯*Cg*1^iii^	0.93	2.96	3.729 (4)	141

Symmetry codes: (i) 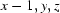; (ii) 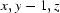; (iii) 
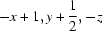
.

**Fig. 1.**
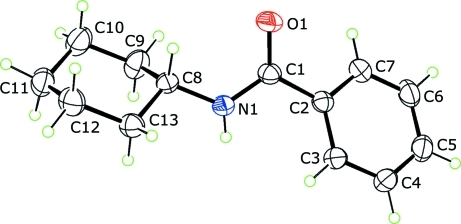
The molecular structure of (I) showing displacement ellipsoids at the 35% probability level.

**Fig. 2.**
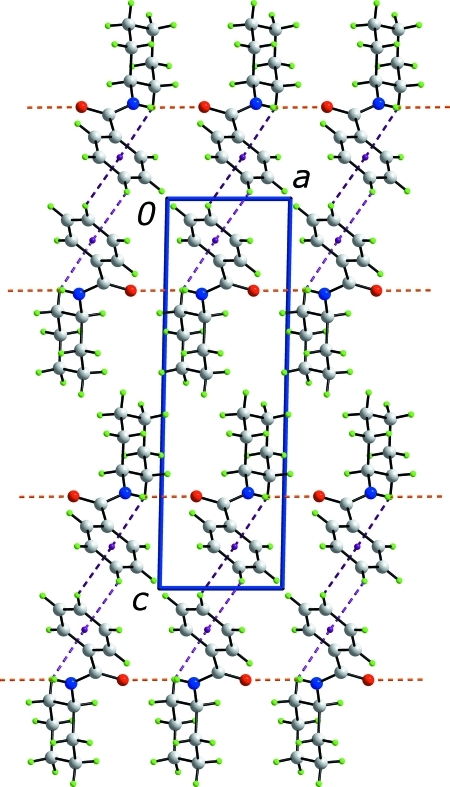
A view in projection down the b axis of the unit-cell contents for (I), highlighting the formation of layers in the ab plane. The N–H···O and C–H···π interactions are shown as orange and purple dashed lines, respectively. Colour code: O, red; N, blue; C, grey; and H, green.

## supplementary crystallographic information

### Comment

Benzamides are frequently used in the synthesis of new and potent anti-convulsant agents (Clark *et al.*, 1988; Leander *et al.*, 1988; Diouf *et al.*, 1997). The structure of the title compound, (I), a benzamide derivative, is reported herein (Fig. 1).

The benzene ring, adjacent to the carbonyl group, twisted with respect to the plane formed through the central amide group; the N1–C1–C2–C3 torsion angle = -30.8 (4) °. In the same way, the putative mirror plane through the cyclohexyl ring (having a chair conformation) is twisted away from the central plane; the O1–N1–C8–C11 torsion angle is 151.3 (4) °. The anti-disposition of the NH and carbonyl groups allows for the formation of N–H···O hydrogen bonds which leads to the formation supramolecular chains aligned along the *a* axis, Fig. 2 and Table 1. These are connected into layers in the *ab* plane *via* C–H···π interactions, Fig. 2 and Table 1.

### Experimental

A solution of cyclohexyl amine (0.458 µl, 4 mmol) in dichloromethane (15 ml) was treated dropwise with benzoyl chloride (0.463 µl, 4 mmol) in the presence of triethanol amine (5 ml) as a catalyst. The resulting mixture was stirred for 1 h. The precipitates that formed were filtered, dried and crystallized from methanol to yield colourless blocks of (I).

### Refinement

The C-bound H atoms were geometrically placed (C–H = 0.93–0.98 Å) and refined as riding with *U_iso_*(H) = 1.2*U_eq_*(C). The N-bound H atom was refined freely. In the absence of significant anomalous scattering effects, 1130 Friedel pairs were averaged in the final refinement.

### Crystal data

**Table:**

C_13_H_17_NO	*Z* = 2
*M**_r_* = 203.28	*F*(000) = 220
Monoclinic, *P*2_1_	*D*_x_ = 1.179 Mg m^−^^3^
Hall symbol: P 2yb	Mo *K*α radiation, λ = 0.71073 Å
*a* = 5.2372 (3) Å	µ = 0.07 mm^−^^1^
*b* = 6.5841 (4) Å	*T* = 293 K
*c* = 16.6029 (12) Å	Block, colourless
β = 91.176 (2)°	0.28 × 0.17 × 0.12 mm
*V* = 572.38 (6) Å^3^	

### Data collection

**Table:**

Bruker APEXII CCD diffractometer	1105 reflections with *I* > 2σ(*I*)
Radiation source: fine-focus sealed tube	*R*_int_ = 0.033
graphite	θ_max_ = 27.5°, θ_min_ = 1.2°
φ and ω scans	*h* = −6→6
5479 measured reflections	*k* = −8→8
1423 independent reflections	*l* = −21→21

### Refinement

**Table:**

Refinement on *F*^2^	Secondary atom site location: difference Fourier map
Least-squares matrix: full	Hydrogen site location: inferred from neighbouring sites
*R*[*F*^2^ > 2σ(*F*^2^)] = 0.045	H atoms treated by a mixture of independent and constrained refinement
*wR*(*F*^2^) = 0.163	*w* = 1/[σ^2^(*F*_o_^2^) + (0.1083*P*)^2^] where *P* = (*F*_o_^2^ + 2*F*_c_^2^)/3
*S* = 1.07	(Δ/σ)_max_ < 0.001
1423 reflections	Δρ_max_ = 0.22 e Å^−^^3^
140 parameters	Δρ_min_ = −0.21 e Å^−^^3^
1 restraint	Absolute structure: unk
Primary atom site location: structure-invariant direct methods	

### Special details

**Table:**

Geometry. All s.u.'s (except the s.u. in the dihedral angle between two l.s. planes) are estimated using the full covariance matrix. The cell s.u.'s are taken into account individually in the estimation of s.u.'s in distances, angles and torsion angles; correlations between s.u.'s in cell parameters are only used when they are defined by crystal symmetry. An approximate (isotropic) treatment of cell s.u.'s is used for estimating s.u.'s involving l.s. planes.
Refinement. Refinement of *F*^2^ against ALL reflections. The weighted *R*-factor *wR* and goodness of fit *S* are based on *F*^2^, conventional *R*-factors *R* are based on *F*, with *F* set to zero for negative *F*^2^. The threshold expression of *F*^2^ > 2σ(*F*^2^) is used only for calculating *R*-factors(gt) *etc*. and is not relevant to the choice of reflections for refinement. *R*-factors based on *F*^2^ are statistically about twice as large as those based on *F*, and *R*- factors based on ALL data will be even larger.

### Fractional atomic coordinates and isotropic or equivalent isotropic displacement parameters (Å^2^)

**Table:**

	*x*	*y*	*z*	*U*_iso_*/*U*_eq_	
O1	0.7158 (3)	0.9885 (4)	0.23591 (14)	0.0636 (8)	
N1	0.2952 (5)	0.9238 (4)	0.24441 (15)	0.0424 (6)	
H1n	0.157 (6)	0.969 (6)	0.2345 (18)	0.045 (9)*	
C1	0.4923 (5)	1.0267 (4)	0.21584 (18)	0.0415 (7)	
C2	0.4362 (5)	1.1979 (5)	0.15919 (15)	0.0381 (6)	
C3	0.2245 (5)	1.2004 (6)	0.10682 (16)	0.0460 (7)	
H3	0.1077	1.0941	0.1071	0.055*	
C4	0.1894 (6)	1.3614 (7)	0.05468 (18)	0.0575 (9)	
H4	0.0488	1.3620	0.0195	0.069*	
C5	0.3571 (6)	1.5201 (6)	0.05375 (19)	0.0602 (10)	
H5	0.3321	1.6270	0.0179	0.072*	
C6	0.5654 (6)	1.5203 (6)	0.1069 (2)	0.0586 (9)	
H6	0.6785	1.6292	0.1076	0.070*	
C7	0.6038 (6)	1.3593 (6)	0.15818 (19)	0.0495 (8)	
H7	0.7455	1.3591	0.1929	0.059*	
C8	0.3233 (5)	0.7589 (4)	0.30240 (17)	0.0405 (7)	
H8	0.4904	0.6958	0.2945	0.049*	
C9	0.3184 (8)	0.8399 (6)	0.3885 (2)	0.0611 (9)	
H9A	0.4570	0.9358	0.3968	0.073*	
H9B	0.1588	0.9108	0.3969	0.073*	
C10	0.3452 (8)	0.6680 (7)	0.4484 (2)	0.0683 (11)	
H10A	0.5140	0.6088	0.4443	0.082*	
H10B	0.3296	0.7218	0.5024	0.082*	
C11	0.1481 (6)	0.5051 (6)	0.4349 (2)	0.0636 (10)	
H11A	−0.0198	0.5593	0.4461	0.076*	
H11B	0.1814	0.3937	0.4719	0.076*	
C12	0.1507 (7)	0.4266 (6)	0.3493 (2)	0.0620 (9)	
H12A	0.0120	0.3306	0.3413	0.074*	
H12B	0.3100	0.3556	0.3405	0.074*	
C13	0.1224 (6)	0.5985 (5)	0.2888 (2)	0.0482 (8)	
H13A	0.1362	0.5444	0.2347	0.058*	
H13B	−0.0455	0.6591	0.2934	0.058*	

### Atomic displacement parameters (Å^2^)

**Table:**

	*U*^11^	*U*^22^	*U*^33^	*U*^12^	*U*^13^	*U*^23^
O1	0.0330 (10)	0.0681 (17)	0.0893 (17)	0.0023 (11)	−0.0042 (10)	0.0243 (15)
N1	0.0333 (12)	0.0437 (14)	0.0500 (14)	0.0042 (11)	−0.0005 (9)	0.0077 (11)
C1	0.0359 (13)	0.0405 (16)	0.0479 (15)	0.0036 (13)	0.0009 (11)	−0.0004 (13)
C2	0.0356 (12)	0.0421 (15)	0.0368 (14)	0.0029 (12)	0.0050 (10)	−0.0031 (13)
C3	0.0398 (14)	0.0546 (18)	0.0436 (15)	0.0001 (14)	−0.0021 (11)	0.0020 (16)
C4	0.0471 (16)	0.080 (2)	0.0453 (16)	0.0045 (18)	−0.0018 (13)	0.0133 (19)
C5	0.0554 (17)	0.067 (2)	0.059 (2)	0.0138 (18)	0.0137 (15)	0.024 (2)
C6	0.0552 (17)	0.0512 (19)	0.070 (2)	−0.0060 (17)	0.0147 (15)	0.0140 (18)
C7	0.0426 (14)	0.0538 (19)	0.0523 (17)	−0.0035 (14)	0.0059 (12)	0.0047 (16)
C8	0.0354 (12)	0.0391 (15)	0.0470 (16)	0.0060 (12)	0.0010 (11)	0.0046 (13)
C9	0.084 (2)	0.051 (2)	0.0476 (17)	−0.0102 (19)	−0.0115 (16)	−0.0008 (16)
C10	0.082 (2)	0.071 (3)	0.0513 (19)	−0.001 (2)	−0.0104 (17)	0.010 (2)
C11	0.0624 (19)	0.062 (2)	0.067 (2)	0.010 (2)	0.0116 (16)	0.022 (2)
C12	0.0633 (19)	0.0396 (18)	0.083 (3)	−0.0053 (16)	0.0001 (17)	0.0068 (18)
C13	0.0457 (15)	0.0427 (17)	0.0560 (19)	−0.0010 (14)	−0.0021 (13)	−0.0011 (15)

### Geometric parameters (Å, °)

**Table:**

O1—C1	1.236 (3)	C8—C9	1.526 (4)
N1—C1	1.331 (4)	C8—H8	0.9800
N1—C8	1.456 (4)	C9—C10	1.511 (5)
N1—H1n	0.80 (3)	C9—H9A	0.9700
C1—C2	1.493 (4)	C9—H9B	0.9700
C2—C7	1.379 (4)	C10—C11	1.502 (5)
C2—C3	1.395 (4)	C10—H10A	0.9700
C3—C4	1.379 (5)	C10—H10B	0.9700
C3—H3	0.9300	C11—C12	1.512 (5)
C4—C5	1.365 (5)	C11—H11A	0.9700
C4—H4	0.9300	C11—H11B	0.9700
C5—C6	1.389 (5)	C12—C13	1.519 (5)
C5—H5	0.9300	C12—H12A	0.9700
C6—C7	1.372 (5)	C12—H12B	0.9700
C6—H6	0.9300	C13—H13A	0.9700
C7—H7	0.9300	C13—H13B	0.9700
C8—C13	1.505 (4)		
			
C1—N1—C8	123.1 (2)	C10—C9—C8	110.6 (3)
C1—N1—H1N	116 (3)	C10—C9—H9A	109.5
C8—N1—H1N	120 (2)	C8—C9—H9A	109.5
O1—C1—N1	122.5 (3)	C10—C9—H9B	109.5
O1—C1—C2	119.8 (2)	C8—C9—H9B	109.5
N1—C1—C2	117.7 (2)	H9A—C9—H9B	108.1
C7—C2—C3	118.8 (3)	C11—C10—C9	112.5 (3)
C7—C2—C1	118.2 (2)	C11—C10—H10A	109.1
C3—C2—C1	123.0 (3)	C9—C10—H10A	109.1
C4—C3—C2	119.7 (3)	C11—C10—H10B	109.1
C4—C3—H3	120.2	C9—C10—H10B	109.1
C2—C3—H3	120.2	H10A—C10—H10B	107.8
C5—C4—C3	121.2 (3)	C10—C11—C12	111.4 (3)
C5—C4—H4	119.4	C10—C11—H11A	109.4
C3—C4—H4	119.4	C12—C11—H11A	109.4
C4—C5—C6	119.4 (3)	C10—C11—H11B	109.4
C4—C5—H5	120.3	C12—C11—H11B	109.4
C6—C5—H5	120.3	H11A—C11—H11B	108.0
C7—C6—C5	119.8 (3)	C11—C12—C13	111.4 (3)
C7—C6—H6	120.1	C11—C12—H12A	109.4
C5—C6—H6	120.1	C13—C12—H12A	109.4
C6—C7—C2	121.2 (3)	C11—C12—H12B	109.4
C6—C7—H7	119.4	C13—C12—H12B	109.4
C2—C7—H7	119.4	H12A—C12—H12B	108.0
N1—C8—C13	111.3 (2)	C8—C13—C12	111.4 (2)
N1—C8—C9	110.8 (3)	C8—C13—H13A	109.3
C13—C8—C9	111.1 (3)	C12—C13—H13A	109.3
N1—C8—H8	107.8	C8—C13—H13B	109.3
C13—C8—H8	107.8	C12—C13—H13B	109.3
C9—C8—H8	107.8	H13A—C13—H13B	108.0
			
C8—N1—C1—O1	0.7 (5)	C3—C2—C7—C6	−0.1 (4)
C8—N1—C1—C2	−177.6 (2)	C1—C2—C7—C6	179.2 (3)
O1—C1—C2—C7	−28.3 (4)	C1—N1—C8—C13	−146.5 (3)
N1—C1—C2—C7	150.1 (3)	C1—N1—C8—C9	89.3 (3)
O1—C1—C2—C3	150.9 (3)	N1—C8—C9—C10	179.5 (3)
N1—C1—C2—C3	−30.8 (4)	C13—C8—C9—C10	55.2 (3)
C7—C2—C3—C4	1.0 (4)	C8—C9—C10—C11	−54.8 (4)
C1—C2—C3—C4	−178.2 (3)	C9—C10—C11—C12	54.6 (4)
C2—C3—C4—C5	−0.6 (5)	C10—C11—C12—C13	−54.1 (4)
C3—C4—C5—C6	−0.7 (5)	N1—C8—C13—C12	−179.8 (3)
C4—C5—C6—C7	1.6 (5)	C9—C8—C13—C12	−55.8 (3)
C5—C6—C7—C2	−1.2 (5)	C11—C12—C13—C8	55.2 (4)

### Hydrogen-bond geometry (Å, °)

**Table:**

Cg1 is the centroid of the C2–C7 ring.

**Table:**

*D*—H···*A*	*D*—H	H···*A*	*D*···*A*	*D*—H···*A*
N1—H1n···O1^i^	0.80 (3)	2.32 (3)	3.065 (3)	157 (3)
C13—H13a···Cg1^ii^	0.97	2.82	3.722 (4)	154
C5—H5···Cg1^iii^	0.93	2.96	3.729 (4)	141

Symmetry codes: (i) *x*−1, *y*, *z*; (ii) *x*, *y*−1, *z*; (iii) −*x*+1, *y*+1/2, −*z*.
